# Report of Eighteen Cases of Pemphigus

**Published:** 1854-05

**Authors:** 


### RECORD OF MEDICAL SCIENCE.

*Report of Eighteen Cases of Pemphigus.* Under the care of Dr. BENNETT, Mr. SKEY, and Mr. STARTIN.

In England, pemphigus is a disease of considerable rarity. It is one, also, which, in many of its forms, is extremely obstinate and difficult to cure. It has yet found no monographist, and has, indeed, received but little attention, the whole of our published information respecting it consisting in a few scattered reports of cases, and in compilations from continental writers. The attempt, therefore, to bring together, in a short report, most of the cases that have recently come under our notice in the London Hospitals, may, it is hoped, not be found fruitless. As might be expected, a large majority of these cases have occurred at the Hospital for Skin Diseases. The following particulars of several of them (Cases 1, 3, 5, 6 and 7) are from notes kindly supplied to us by Mr. Tyler, a gentleman to whose labors in dermatology the pages of the *Medical Times and Gazette* have already, on more than one occasion, been indebted; the others are from our own.

Pemphigus (*the bullous disease,*—from *pemphis,* a bleb) is a disease about the diagnosis of which no difficulty exists, since any one who has either seen a case, or a good portrait of it, would be quite unable to confuse its features with those of any other. It was formerly separated into two varieties, under the names of pemphigus and pompholyx; but, as the distinction between them was founded merely on the different degree of chronicity, it has latterly been very properly discarded. The way, however, in which pemphigus has been divided by late writers, following Rayer, into *acute* and *chronic*, is also liable to objection, since the so-called *acute* cases are almost invariably simply transient and benign, all the characters of severity, whether constitutional or local, being reserved for those of the chronic class. The subjects in connexion with the disease especially requiring elucidation are,—1st. The age, condition of health, etc., most liable to its attacks; 2ndly. The nature of the attendant symptoms; 3rdly. The predisposing causes; and 4thly. The treatment. [We omit the cases.]

*Conclusions.*—Examination of the more detailed notes before us, from which the preceding cases have been compiled, appears to warrant the following conclusions:—

1. That pemphigus is a disease affecting all periods of life; especially liable to occur between the ages of 4 and 25.

2. That, like many other skin diseases, it very frequently recurs in those who have once been its subjects.

3. That it usually affects those only of a fair complexion and thin skin. (To this we find no exception among the cases in which note has been made as to the complexion.)

4. That it is rather more common in the male than the female sex (10 to 8.)

5. That its severe chronic and relapsing forms are more frequent than the benign and transient.

6. That the parts most liable to be affected are the legs, arms, genitals, abdomen; seldom the face, and very rarely the hairy scalp.

7. That the serum of the bullæ is almost always alkaline. (It was tested in most of the cases, and no exception occurred; the alkalinity was generally very great.)

8. That it is very rarely a symptom of congenital, and perhaps never of acquired syphilis.

9. That it occurs commonly to those of good physical conformation, but is mostly coincident with temporary cachexia.

10. That is is not very markedly influenced by season.

11. That its idiopathic infantile form is a very mild disease, and will usually recover spontaneously.

12. That it is not, as a rule, associated with any particular form of cachexia. (In but two of the above cases were the patients scrofulous; none were known to be rheumatic, or to have had ague; dyspepsia was an attendant in but few.)

13. That the general indications are for the use of tonic regimen and generous diet, (Cases 13 and 14;) but that these will not suffice for the cure (Cases 3, 4 and 14).

14. That arsenic may be esteemed an almost specific remedy even in the worst class of cases, (2, 3, 4, 14, etc. etc.) [The careful perusal of the preceding series will, we think, convince the reader that this proposition is not too strongly put. Many of the patients were, when admitted under Mr. Startin's care, in a truly deplorable condition,—the disease had produced extreme irritation, it had lasted for many months or years, it had resisted all sorts of treatment previously. In every instance but two (Cases 3 and 15) the most marked benefit attended the adoption of the arsenical plan.]

15. That arsenic does not merely repress the eruption, but remedies the unknown constitutional cause on which that symptom depends, always very much benefitting the general health of the patient.

16. That arsenic does not prevent the liability to subsequent attacks, but that such attacks are always much less severe than the original one, and tend, if treated by the same remedy, to diminish in intensity on each successive occasion.

17. That the early age of the patient does not in the least forbid resort to the arsenical treatment.

Those acquainted with the literature of this subject will observe, that the above conclusions differ considerably in some respects from the statements to be found in books. The disease itself may probably differ somewhat in London and on the Continent. Gilibert, whose monograph on it is the best extant, appears, for instance, to have met with but three examples of chronic pemphigus, all of them in elderly and enfeebled subjects. A current opinion has accordingly prevailed, that that form is almost peculiar to the aged, while the fact is, as we have above shown, that in London, at any rate, the young are much more frequently its subjects. Cases of relapsing pemphigus, or those in which the disease has extended over many years, do not appear to have attracted much notice from previous writers. Pemphigus is not known to prevail endemically in any part of England ; on the contrary, it seems to be about equally scattered over all districts. Two cases came under our notice in York some years ago, and during the last summer we saw a very well-marked case in the Leeds Infirmary, under the care of Mr. Samuel Hey. Mr. Hey informed us, that the disease was very rare in Leeds, and that he had, during many years, seen but that one example. There does not appear to be much foundation for the opinion that the disease prevails most in damp localities, and on the banks of rivers. Such a notion is supported by but a small proportion of the above cases. The preceding series probably scarcely presents the benign and chronic forms in their due proportions as to frequency of occurrence, since cases of the former are often of so transient a character that they never come under care at Hospitals. With regard to the treatment of the chronic form by arsenic, we have recorded *all* that we have seen. A case has been mentioned to us, however, by a gentleman of very careful observation, in which the arsenic is stated to have quite failed to cure the disease, while it seriously interfered with the patient's (a child) health. We have not obtained particulars as to administration, etc.

The disease known in London as Rupia escharotica, but described by Dr. Corrigan as a form of infantile pemphigus, has been altogether omitted in the above, and is reserved for a future report. Whatever may be said of its primary stage, its after-course has no sort of resemblance to pemphigus.—*Med. Times and Gazette.*

